# Dental fear and its possible relationship with periodontal status in Chinese adults: a preliminary study

**DOI:** 10.1186/1472-6831-15-18

**Published:** 2015-01-28

**Authors:** Yeungyeung Liu, Xin Huang, Yuxia Yan, Hanxiao Lin, Jincai Zhang, Dongying Xuan

**Affiliations:** Department of Periodontology, Guangdong Provincial Stomatological Hospital, Southern Medical University, S366 Jiangnan Boulevard, 510280 Guangzhou, China; Department of Statistics, Southern Medical University, Guangzhou, China; Department of Endocrinology, Zhujiang Hospital of Southern Medical University, Guangzhou, China

**Keywords:** Dental fear, Periodontal disease, Oral health behaviors

## Abstract

**Background:**

The aim of the present study was to describe the characteristics of dental fear of Chinese adult patients with periodontal disease and provide information for clinical assessment.

**Methods:**

A total of 1203 dental patients completed questionnaires that included Corach’s Dental Anxiety Scales (DAS), Dental Fear Survey (DFS) and the short-form Dental Anxiety Inventory (S-DAI). Among all the patients, 366 cases were self-reported periodontal disease. The general characteristics were described, such as socio-demographics, dental attendances and oral health behaviors. The statistical analysis was performed by t-test, Mann–Whitney U test and linear regression respectively to evaluate correlations between dental fear and general characteristics according to the three scales.

**Results:**

The prevalence of dental fear was 74% among 1203 patients, 23.4% of total with high dental fear, while 27.3% in the patients with periodontal disease. The average score of DAS and DFS for patients with periodontal disease was significantly higher than those without periodontal disease. The regression analysis indicated that gender, age, periodontal status, dental attendances and oral health behaviors were correlated with dental fear. Among 366 patients with periodontal disease, gender, dental attendances and oral health behaviors had correlation with dental fear. The analysis of DFS scale exhibited that ‘drilling with handpiece’ and ‘injecting the anesthetic’ were the most important factors to contribute to dental fear.

**Conclusions:**

There was high prevalence of dental fear in Chinese adult patients, particularly in patients with periodontal disease, and high level of dental fear may lead to poor periodontal status.

## Background

Dental fear is a significant public and oral health issue. Extreme dental fear affects a wide portion of population [1–3]. In previous studies, dental fear was related to less frequent dental visiting, poorer oral health and greater functional impairment [4–7].

The etiological factors of dental fear included negative information, witnessing or having bad experience and negative condition. Meanwhile, dental fear was regarded as a complex process with simultaneous interaction of both exogenous and endogenous factors. Exogenous components were involved in direct or indirect learning from adverse experience, and endogenous components were more likely to be genetically determined and physiological in nature [8]. It was widely accepted that most of patients with dental fear had painful treatment experience. High pretreatment fear level had been reported for pain experience during periodontal scaling/non-surgical periodontal treatment [9, 10].

Several previous studies found that periodontal therapy was associated with high level of dental fear. About 71% patients had dental fear related to periodontal therapy, and 12.1% patients had extreme anticipatory fear during treatment [11, 12]. Fear of treatment could affect patient compliance and result in deterioration of the periodontal health [13]. Periodontal therapy consisted of multiple long treatment sessions, exposing the patients to the feared situation. Continuous high level of dental fear caused by experiencing discomfort or pain in treatment process might have negative effects on clinical outcome. Furthermore, the patients with periodontal disease used to worry about dental treatment, which exerted the negative effect on oral hygiene condition.

Periodontal disease is one of the two most important oral diseases [14], which is highly prevalent worldwide and represents a major public health problem in many countries. With 80%-90% incidence in Chinese adult, periodontitis was the major cause of tooth loss. About 15%-20% tooth loss were caused by severe periodontitis at the age of 35–44 according to World Health Organization (WHO). To date, only a handful of studies have reported dental fear of patients with periodontal disease [11, 12]. Nevertheless, there is lack of assessment of dental fear in Chinese adult population with and without periodontal disease. The present study used validated questionnaires to assess the prevalence of dental fear in Chinese adult patients, and to evaluate the potential effect of periodontal status on dental fear.

## Methods

### Patients’ recruitment

The patients, who presented from July 2013 to November 2013 at the Outpatient Clinics of Guangdong Provincial Stomatological Hospital, University of Southern Medical, were randomly recruited as subjects to perform the questionnaires. Written informed consent for participation was obtained from all participants prior to the investigation. The present study was approved by the Ethical Committee, Guangdong Provincial Stomatological Hospital, University of Southern Medical.

Inclusion criteria for the subjects included: 1. Over 18 years old; 2. No cognitive impairments and eye diseases; 3. Able to complete the questionnaire independently. Exclusion criteria for the subjects included: 1. History of mental illness; 2. Illiteracy and noncooperation; 3. Anxiolytic, sedative or analgesic agent took 3 day before the survey.

### Data collection and questionnaires

A total of 1283 questionnaires were collected, of which 1203 were validated. Among the 1203 patients, 366 were self-reported periodontal disease. The self-reported periodontal disease was assessed according to the three items described in the literatures [15, 16]: “Do you have a loose tooth? (Yes/No)”; “Have you had periodontal disease with bone loss? (Yes/No)”; “Has your dentist/hygienist told you that you have deep pocket? (Yes/No)”. Patients who had any affirmative responses for the three questions were considered as patients with periodontal disease.

The questionnaire included 3 separated tests: Corach’s Dental Anxiety Scales (DAS), Dental Fear Survey (DFS), and the short-form Dental Anxiety Inventory (S-DAI), and was composed of 33 multiple-choice questions in total. The DAS presents four questions related to concerns about visiting the dentist [17], with the first two questions relating to general anxiety and the second two questions relating to anticipated fear of specific stimuli. A score of 13 or greater on the DAS was defined as “high dental fear”, as widely accepted previously [18]. The DAS was the most widely used dental fear scale for adults, which reported good reliability and validity. However, it failed to provide additional information regarding what the patient specifically fears. Therefore, the Dental Fear Survey (DFS) was also applied for compensation, which consisted of 20 items with five alternative answers to each, rating from high to low intensity [19, 20]. The S-DAI with 9 items, which was established by Stouthard et al. [21], was also used to assess physical reactions, thoughts and behavioral aspects of dental fear experienced by the individual [22]. The DFS and S-DAI both had good reliability and validity tested Chinese version [23, 24].

General information besides questionnaire contained dental attendances (regular or irregular), past dental visit and socio-demographic features including gender, age, education, marital status, smoking and alcohol use. Oral health behaviors were investigated by three questions: “Do you have bleeding on brushing?”; “Do you feel your teeth sensitive?”; “Do you have regular scaling?”.

### Data analysis

T-test was used to analyze the differences of prevalence of dental fear between the patients with and without periodontal disease. Mann–Whitney U test was used to analyze the differences of the percentage of DAS evaluation between patients with and without periodontal disease. Linear regression was used to analyze the correlation between the patients’ general characteristics and dental fear. For all statistical analysis, *P* values were two-tailed and level of significance was set at *P* = 0.05.

## Results

A total of 1283 patients attended the survey, and 1203 questionnaires were validated. Among the 1203 patients, there were 438 male (36.24%) and 765 female (63.76%), and the average age was 37.7 ± 14.8 (range from 18 to 78), 366 subjects were self-reported periodontal disease. Among the periodontal cases, there were 131(35.8%) male and 232 female (63.4%), and the average age was 42.4 ± 14.6 years (range from 18 to 78).

The results of regression analysis exhibited the relationship between dental fear and socio-demographic variables among 1203 patients as shown in Table 1. The related factors that affected dental fear were gender, age, periodontal status, dental attendances, scaling, bleeding on brushing and dentine hypersensitivity. The prevalence of dental fear was significantly higher in female (*P* < 0.001). The dental fear was negatively related with age. For oral health behaviors aspect, dental fear of ‘regular scaling’ was significantly lower than ‘scaling occasionally’ and ‘never scaling’. Patients who had ‘always bleeding on brushing’ and ‘always dentine hypersensitivity’ had significantly higher level of dental fear than ‘never bleeding on brushing’ and ‘never dentine hypersensitivity’. The results also exhibited that dental fear was not related with past dental visit, smoking and alcohol use.

**Table 1:** **Results of linear regression analysis in three scales among 1203 patients**

	Gender	Age	Marital status	Education	Periodontal status	Dental attendances	Scaling	Bleeding on brushing	Dentine hypersensitivity
**DAS**									
B	1.44	-0.03	0.68	0.28	0.56	-0.45	0.43	0.38	0.53
Beta	0.23	-0.15	0.11	0.06	0.08	-0.06	0.10	0.07	0.10
*P*	<0.001	<0.001	0.003	0.034	0.004	0.045	0.002	0.013	0.001
95% CI	1.09 ~ 1.79	-0.05 ~ -0.01	0.23 ~ 1.12	0.22 ~ 0.54	0.17 ~ 0.94	-0.89 ~ -0.01	0.15 ~ 0.71	0.08 ~ 0.68	0.23 ~ 0.84
**DFS**									
B	8.13	-0.09	NO	1.80	3.49	-2.44	2.38	2.68	2.27
Beta	0.25	-0.08	NO	0.07	0.10	-0.06	0.10	0.10	0.08
*P*	<0.001	0.010	NO	0.009	0.001	0.036	0.001	0.001	0.005
95% CI	6.33 ~ 9.33	-0.15 ~ -0.02	NO	0.45 ~ 3.14	1.51 ~ 5.46	-4.71 ~ -0.16	0.93 ~ 3.82	1.13 ~ 4.22	0.69 ~ 3.85
**S-DAI**									
B	3.56	-0.04	NO	NO	1.41	NO	1.39	NO	NO
Beta	0.20	-0.07	NO	NO	0.08	NO	0.11	NO	NO
*P*	<0.001	0.030	NO	NO	0.012	NO	<0.001	NO	NO
95% CI	2.58 ~ 4.55	-0.07 ~ 0.00	NO	NO	0.31 ~ 2.51	NO	0.64 ~ 2.13	NO	NO

B, Unstandardized Coefficients; Beta, Standardized coefficients; 95% CI, 95% Confidence interval; NO, variable not included in the model.

The dental fear of patients with periodontal disease was significantly higher (10.70 ± 3.09) than those without periodontal disease (10.24 ± 3.00) according to the result of DAS. In DFS evaluation, the dental fear level of patients with periodontal disease was 49.80 ± 15.80, which significantly higher than those without periodontal disease (46.91 ± 15.72). There were no statistical significances of dental fear level between patients with (23.36 ± 8.51) and without (22.63 ± 8.59) periodontal disease in S-DAI (Table 2).

**Figure 1:**
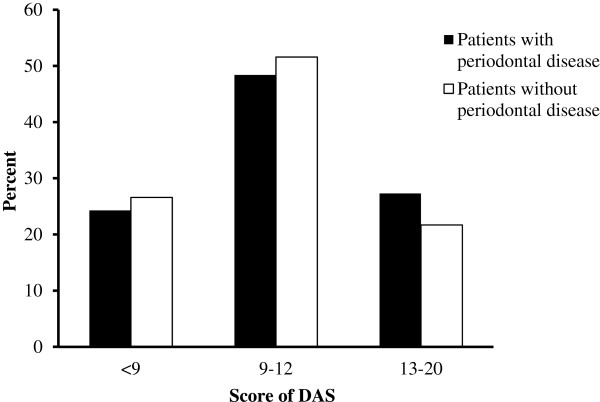
**The percentage of the DAS in patients with and without periodontal disease.** The percentage of high dental fear in patients with periodontal disease was more than those without periodontal disease.

According to the results of DAS evaluation, in patients with periodontal disease, 89 (24.3%) cases scored <9, 177(48.4%) cases 9–12, and 100(27.3%) cases 13–20. The percentages of DAS evaluation in patients with and without periodontal disease were shown in Figure 1. In contrast, in patients without periodontal disease, there were 222(26.6%) patients recorded <9 points, 432(51.6%) patients 9–12, and 182(21.7%) patients 13–20. Comparison of two type patients had significant differences according to DAS evaluation. Different DAS evaluation of the constituent ratio of periodontal disease patients in DAS < 9 was 28.62%, 9–12 was 29%, 13–20 was 35.46%, which increased by dental fear level.

**Table 2:** **Dental fear level of DAS, DFS and S-DAI between patients with and without periodontal disease**

	Patients with periodontal disease, Mean ± SD (n = 366)	Patients without periodontal disease, Mean ± SD (n = 837)	t value	***P***
DAS	10.70 ± 3.09	10.24 ± 3.00	-2.43	0.015
DFS	49.80 ± 15.80	46.91 ± 15.72	-2.88	0.004
S-DAI	23.36 ± 8.51	22.63 ± 8.59	-1.37	0.172

The analysis of dental fear in periodontal disease patients with socio-demographics was presented in Table 3. The related factors that affected dental fear were gender, dental attendances, scaling, bleeding on brushing and dentine hypersensitivity. The results revealed that dental fear was not related with age, education, marital status, past dental visit, smoking and alcohol use in patients with periodontal disease.

**Figure 2:**
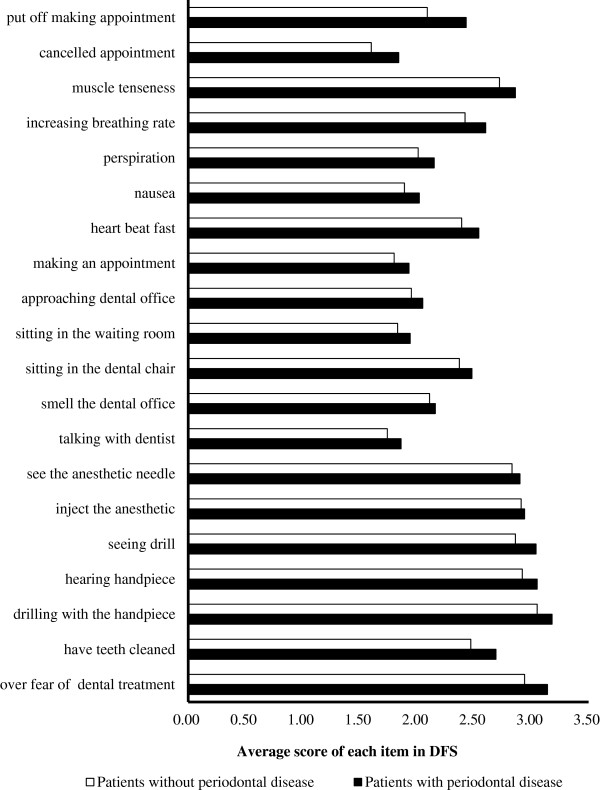
**The average score of each item in DFS between patients with and without periodontal disease.** The five highest items were ‘drilling with the handpiece’, ‘overall fear in dental treatment’, ‘hearing drill’, ‘seeing drill’ and ‘injecting the anesthetic’ in both kinds of patients.

The average score of each item in DFS between patients with and without periodontal disease was shown in Figure 2. The five highest items were ‘tooth drilling’ , ‘overall fear in dental treatment’ , ‘hearing drill’ , ‘seeing drill’ and ‘injecting the anesthetic’.

**Table 3:** **Results of linear regression analysis in three scales of patients with periodontal disease**

	Gender	Dental attendances	Scaling	Bleeding on brushing	Dentine hypersensitivity
**DAS**					
B	1.50	-1.28	NO	0.74	0.75
Beta	0.23	-0.18	NO	0.26	0.14
*P*	<0.001	<0.001	NO	0.005	0.008
95% CI	0.87 ~ 2.14	-1.98 ~ -0.58	NO	0.22 ~ 1.25	0.20 ~ 1.29
**DFS**					
B	7.17	-4.90	3.90	3.07	4.38
Beta	0.22	-0.14	0.16	0.12	0.16
*P*	<0.001	0.015	0.004	0.020	0.002
95% CI	3.98 ~ 10.37	-8.87 ~ -0.94	1.27 ~ 6.53	0.48 ~ 5.66	1.63 ~ 7.13
**S-DAI**					
B	3.55	NO	2.82	NO	NO
Beta	0.20	NO	0.22	NO	NO
*P*	<0.001	NO	<0.001	NO	NO
95% CI	1.77 ~ 5.34	NO	1.52 ~ 4.12	NO	NO

B, Unstandardized Coefficients; Beta, Standardized coefficients; 95% CI, 95% Confidence interval; NO, variable not included in the model.

## Discussion

### The combination of three dental fear scales

Dental fear should be studied with regard to the situation to which it pertains, the reactions it evokes, and its duration [25]. No single scale of dental fear contains various involved aspects. This study used three scales, including DAS, DFS and S-DAI, to evaluate dental fear in different aspects. DFS reflects dental fear informatively to help clinicians understand a patient’s fear better, while DAS measures dental fear generally [26]. S-DAI focuses on psychometric grounds [21], of which nearly half of its items reflect the emotional reactions [26].

In patients with periodontal disease, the dental fear score was significantly higher than patients without periodontal disease according to DAS and DFS, but not in S-DAI. Dental attendances and oral health behaviors were related to dental fear in DAS and DFS, but not in S-DAI. The differences might be caused by the different concentration of the three scales, particularly S-DAI, which focused on psychometric grounds. Researchers drew different conclusions for the same concerned problem from different scales. Malvania et al. reported that age was not related to dental fear according to Modified Dental Anxiety Scale (MDAS) [27], whereas Armfield et al. reported that older adults had significant lower level of dental fear than youths using single item Dental Anxiety Question [28]. In the present study, combination with three scales could enhance the accuracy of the results.

### The prevalence of dental fear in Chinese adult patients

The average score of DAS was 10.38 with 23.4% high dental fear among 1203 Chinese patients, which was higher than other countries. It was 9.0 on DAS with 16.7% high dental fear prevalence in Norway population [29], while 8.4 with 13% high dental fear in British and 18.1% high dental fear in Australian [18, 30].

In the present study, the prevalence of dental fear was significantly higher in women than men, which was consistent with literatures [31–34]. Possible reasons might be related to higher occurrences of anxiety and phobia in female, or even heritability [35, 36].

The present study indicated that age was another factor affecting dental fear as previous [6, 33]. Maggirias et al. reported that younger adults were apt to dental fear [37]. A general clinical impression was that the elders were more tolerant to pain. Moreover, the high anxiety level in young patients could be due to the insufficient experience of the applying instruments, such as needle, handpiece or any other fear invoking equipment.

The patients who had regular dental attendances showed lower level of dental fear in the present study. In previous studies, patients without regular dental care were related to high level of dental fear [8, 29]. Moreover, Bernson et al. reported that dental anxiety was related with dental attendances [8].

### Poor periodontal status may contribute to high dental fear

Periodontal status was an important factor affecting dental fear [11, 12]. Patients with periodontal disease were apt to express high general anxiety. Stress, depression and anxiety were not yet confirmed as definite risk factors of periodontal disease, but considered as potential factors affecting the occurrence, development and prognosis of periodontal disease [35, 38, 39]. Many emerging evidences indicated that periodontal disease was associated with poor mental health. Saletu et al. suggested that anxious emotion should be considered as a relevant pathogenic factor for periodontitis [40]. Additionally, the endogenous aetiologies of dental fear were considered as general anxiety and depressive disorders, especially multiple phobias and depressive personality [41–43].

The present study exhibited correlations between oral health behaviors and dental fear among 1203 patients. Patients without regular scaling expressed significantly higher dental fear [6, 7, 32]. Individuals, who have high dental fear, would delay treatment, and it might lead to more extensive development of disease, which ultimately required more invasive and potentially painful treatment, and these experience might contribute to the increase of dental fear: this is the idea of a ‘vicious cycle’ [5]. The level of dental fear in patients with periodontal disease was higher, and the oral health behaviors including ‘scaling’ , ‘teeth hypersensitive’ and ‘bleeding on brushing’ were related to dental fear in the present study. As a result, the periodontal status would be exacerbated because of high level of dental fear, then resulting in poor oral health status, and the vicious cycle established. Hence, the patients with periodontal disease or poor oral health should be noticed by clinicians to the level of dental fear.

In the present study, self-reported measures were used to identify periodontal disease. Epidemiological studies based on large population were usually conducted by surveys rather than clinical examination for their easy performance, low cost and simple rating systems. Gilbert et al. suggested that loose tooth was significant associated with attachment loss [16]. Moreover, tooth mobility was considered as a valid measure for positive relationship with severe periodontal disease [15]. “Have you had periodontal disease with bone loss?” and “Has your dentist/hygienist told you that you have deep pocket?” had been proved sufficient validity [15]. The three questions mentioned above were used in the present study. Additionally, good validity was shown in other self-reported questions. For example, Eke et al. [44] reported good sensitivity and specificity of the combination of demographic measures and responses effects using 5 self-reported questions for predicting periodontitis.

However, self-reported measures may cause inaccuracy for its patient-base evaluation, which is the limitation of the present study. Therefore, it is necessary to perform clinical examination to diagnosis periodontal disease for further investigation of the relationship between dental fear and periodontal status.

### Specific concerns invoking dental fear in dental treatment

According to DFS evaluation, the five highest fear items were ‘drilling with the handpiece’ , ‘overall fear of dental treatment’ , ‘injecting the anesthetic’ , ‘hearing drill’ and ‘seeing drill’. We found that ‘drilling with the handpiece’ was the most important concern of invoking dental fear. Most negative items were related to tooth drilling, giving that it had long been listed as one of the most fear provoking items in dental office [45, 46]. Oosterink et al. reported that ‘dentist drilling your tooth or molar’ was the seventh most fear-provoking stimuli of 67 potentially stimuli [47]. Another important high fear-provoking item was ‘inject the anesthetic’ as previous [18, 31]. Dental phobia was considered as a specific phobia subtype independently in blood-injection-injury (BII) cluster [48]. Under such condition, patients who feared oral injections were at high level of dental fear and it could led to miss or delay dental appointments [49].

## Conclusions

There was a high prevalence of dental fear in Chinese adult patients, particularly in patients with periodontal disease. Dental attendances and oral health behaviors could have an effect on dental fear, and high level of dental fear may lead to poor periodontal status.

## Abbreviations

DAS: Corach’s Dental Anxiety Scales
DFS: Dental Fear Survey
S-DAI: Short-form Dental Anxiety Inventory
WHO: World Health Organization
VAS: Visual Analogue Scale
B: Unstandardized Coefficients
Beta: Standardized coefficients
CI: Confidence interval
NO: Variable not included in the model.

## References

[1] Meng X, Heft MW, Bradley MM, Lang PJ (2007). Effect of fear on dental utilization behaviors and oral health outcome. Community Dent Oral Epidemiol.

[2] Wogelius P, Poulsen S, Sorensen HT (2003). Prevalence of dental anxiety and behavior management problems among six to eight years old Danish children. Acta Odontol Scand.

[3] Samorodnitzky GR, Levin L (2005). Self-assessed dental status, oral behavior, DMF, and dental anxiety. J Dent Educ.

[4] Armfield JM (2013). Predicting dental avoidance among dentally fearful Australian adults. Eur J Oral Sci.

[5] Armfield JM, Stewart JF, Spencer AJ (2007). The vicious cycle of dental fear: exploring the interplay between oral health, service utilization and dental fear. BMC Oral Health.

[6] Eitner S, Wichmann M, Paulsen A, Holst S (2006). Dental anxiety–an epidemiological study on its clinical correlation and effects on oral health. J Oral Rehabil.

[7] Armfield JM, Slade GD, Spencer AJ (2009). Dental fear and adult oral health in Australia. Community Dent Oral Epidemiol.

[8] Bernson JM, Elfstrom ML, Hakeberg M (2013). Dental coping strategies, general anxiety, and depression among adult patients with dental anxiety but with different dental-attendance patterns. Eur J Oral Sci.

[9] Chung DT, Bogle G, Bernardini M, Stephens D, Riggs ML, Egelberg JH (2003). Pain experienced by patients during periodontal maintenance. J Periodontol.

[10] Guzeldemir E, Toygar HU, Cilasun U (2008). Pain perception and anxiety during scaling in periodontally healthy subjects. J Periodontol.

[11] Fardal O, Johannessen AC, Linden GJ (2001). Pre-treatment conceptions of periodontal disease and treatment in periodontal referrals. J Clin Periodontol.

[12] Fardal O, Hansen BF (2007). Interviewing self-reported highly anxious patients during periodontal treatment. J Periodontol.

[13] Rizzardo R, Borgherini G, Cappelletti L (1991). Illness behaviour and anxiety in dental patients. J Psychosom Res.

[14] Bratthall D, Petersen PE, Stjernsward JR, Brown LJ, Jamison DT, Breman JG, Measham AR, Alleyne G, Claeson M, Evans DB, Jha P, Mills A (2006). Oral and craniofacial diseases and disorders. Disease control priorities in developing countries.

[15] Blicher B, Joshipura K, Eke P (2005). Validation of self-reported periodontal disease: a systematic review. J Dent Res.

[16] Gilbert GH, Litaker MS (2007). Validity of self-reported periodontal status in the Florida dental care study. J Periodontol.

[17] Corah NL (1969). Development of a dental anxiety scale. J Dent Res.

[18] Armfield JM (2010). The extent and nature of dental fear and phobia in Australia. Aust Dent J.

[19] Kleinknecht RA, Klepac RK, Alexander LD (1973). Origins and characteristics of fear of dentistry. J Am Dent Assoc.

[20] Kleinknecht RA, Bernstein DA (1978). The assessment of dental fear. Behav Ther.

[21] Stouthard MEA, Mellenbergh GJ, Hoogstraten J (1993). Assessment of dental anxiety: a facet approach. Anxiety Stress Coping.

[22] Porritt J, Buchanan H, Hall M, Gilchrist F, Marshman Z (2013). Assessing children’s dental anxiety: a systematic review of current measures. Community Dent Oral Epidemiol.

[23] Huan-you LIANG, Zhu-li PENG, Ji-yang PAN, Qian TANG, Ping WANG (2006). Development and Evaluation of Chinese Version of Dental Fear Survey (DFS). J Sun Yat-sen Univ (Medical Sciences).

[24] Ng SK, Stouthard ME, Keung Leung W (2005). Validation of a Chinese version of the dental anxiety inventory. Community Dent Oral Epidemiol.

[25] Schuurs AH, Hoogstraten J (1993). Appraisal of dental anxiety and fear questionnaires: a review. Community Dent Oral Epidemiol.

[26] Armfield JM (2010). How do we measure dental fear and what are we measuring anyway?. Oral Health Prev Dent.

[27] Malvania EA, Ajithkrishnan CG (2011). Prevalence and socio-demographic correlates of dental anxiety among a group of adult patients attending a dental institution in Vadodara city, Gujarat. India Indian J Dent Res.

[28] Armfield JM, Spencer AJ, Stewart JF (2006). Dental fear in Australia: who’s afraid of the dentist?. Aust Dent J.

[29] Astrom AN, Skaret E, Haugejorden O (2011). Dental anxiety and dental attendance among 25-year-olds in Norway: time trends from 1997 to 2007. BMC Oral Health.

[30] Bedi R, McGrath C (2000). Factors associated with dental anxiety among older people in Britain. Gerodontology.

[31] Enkling N, Marwinski G, Johren P (2006). Dental anxiety in a representative sample of residents of a large German city. Clin Oral Investig.

[32] Schuller AA, Willumsen T, Holst D (2003). Are there differences in oral health and oral health behavior between individuals with high and low dental fear?. Community Dent Oral Epidemiol.

[33] Kumar S, Bhargav P, Patel A, Bhati M, Balasubramanyam G, Duraiswamy P (2009). Does dental anxiety influence oral health-related quality of life? Observations from a cross-sectional study among adults in Udaipur district. India J Oral Sci.

[34] Humphris G, Crawford JR, Hill K, Gilbert A, Freeman R (2013). UK population norms for the modified dental anxiety scale with percentile calculator: adult dental health survey 2009 results. BMC Oral Health.

[35] Warren KR, Postolache TT, Groer ME, Pinjari O, Kelly DL, Reynolds MA (2014). Role of chronic stress and depression in periodontal diseases. Periodontol.

[36] Ray J, Boman UW, Bodin L, Berggren U, Lichtenstein P, Broberg AG (2010). Heritability of dental fear. J Dent Res.

[37] Maggirias J, Locker D (2002). Five-year incidence of dental anxiety in an adult population. Community Dent Health.

[38] Vettore MV, Leao AT, Monteiro Da Silva AM, Quintanilha RS, Lamarca GA (2003). The relationship of stress and anxiety with chronic periodontitis. J Clin Periodontol.

[39] Ng SK, Keung Leung W (2006). A community study on the relationship between stress, coping, affective dispositions and periodontal attachment loss. Community Dent Oral Epidemiol.

[40] Saletu A, Pirker-Frühauf H, Saletu F, Linzmayer L, Anderer P, Matejka M (2005). Controlled clinical and psychometric studies on the relation between periodontitis and depressive mood. J Clin Periodontol.

[41] Locker D, Poulton R, Thomson WM (2001). Psychological disorders and dental anxiety in a young adult population. Community Dent Oral Epidemiol.

[42] Armfield JM, Slade GD, Spencer AJ (2008). Cognitive vulnerability and dental fear. BMC Oral Health.

[43] Oosterink FM, De Jongh A, Hoogstraten J, Aartman IH (2008). The Level of Exposure-Dental Experiences Questionnaire (LOE-DEQ): a measure of severity of exposure to distressing dental events. Eur J Oral Sci.

[44] Eke PI, Dye BA, Wei L, Slade GD, Thornton-Evans GO, Beck JD (2013). Self-reported 31 measures for surveillance of periodontitis. J Dent Res.

[45] Gale EN (1972). Fears of the dental situation. J Dent Res.

[46] Milgrom P, Fiset L, Melnick S, Weinstein P (1988). The prevalence and practice management consequences of dental fear in a major US city. J Am Dent Assoc.

[47] Oosterink FM, De Jongh A, Aartman IH (2008). What are people afraid of during dental treatment? Anxiety-provoking capacity of 67 stimuli characteristic of the dental setting. Eur J Oral Sci.

[48] Van Houtem CM, Aartman IH, Boomsma DI, Ligthart L, Visscher CM, De Jongh A (2014). Is Dental Phobia a Blood-Injection-Injury Phobia?. Depress Anxiety.

[49] McDonald F, Robinson PD PFT (2000). Local anesthesia in dentistry.

[CR50] The pre-publication history for this paper can be accessed here: http://www.biomedcentral.com/1472-6831/15/18/prepub

